# Seasonal Effects of Constructed Wetlands on Water Quality Characteristics in Jinshan Lake: A Gate Dam Lake (Zhenjiang City, China)

**DOI:** 10.3390/biology13080593

**Published:** 2024-08-06

**Authors:** Xiao Li, Xinlin Liu, Yulong Zhang, Jing Liu, Yang Huang, Jian Li

**Affiliations:** 1 ART School, Jiangsu University, Zhenjiang 212013, China; 1000002936@ujs.edu.cn (X.L.); hale971130@163.com (Y.H.); 2 Institute of International Education, New Era University College, Kajang 43000, Malaysia; 3 School of the Environment and Safety Engineering, Jiangsu University, Zhenjiang 212013, China; 15680961767@163.com (X.L.); zyl@stmail.ujs.edu.cn (Y.Z.); 3220904002@stmail.ujs.edu.cn (J.L.)

**Keywords:** constructed wetland, seasonal influence, water depth, water quality, nitrogen, phosphorus, chlorophyll-a

## Abstract

**Simple Summary:**

In this study, we investigated the seasonal effects of constructed wetlands on water quality characteristics in a gate dam lake. The research reveals pronounced seasonal changes in the lake’s water environment index. During winter, nitrate nitrogen and BOD_5_ interact significantly, jointly becoming the primary factors restricting lake water quality. In summer, the constructed wetlands effectively improve TN levels. Furthermore, water depth plays a regulatory role in Chl-a content, ammonia–nitrogen, and nitrite–nitrogen by influencing light penetration and water temperature.

**Abstract:**

Urban lakes commonly suffer from nutrient over-enrichment, resulting in water quality deterioration and eutrophication. Constructed wetlands are widely employed for ecological restoration in such lakes but their efficacy in water purification noticeably fluctuates with the seasons. This study takes the constructed wetland of Jinshan Lake as an example. By analyzing the water quality parameters at three depths during both summer and winter, this study explores the influence of the constructed wetland on the water quality of each layer during different seasons and elucidates the potential mechanisms underlying these seasonal effects. The results indicate that the constructed wetland significantly enhances total nitrogen (TN) concentration during summer and exhibits the capacity for nitrate–nitrogen removal in winter. However, its efficacy in removing total phosphorus (TP) is limited, and may even serve as a potential phosphorus (P) source for the lake during winter. Water quality test results of different samples indicated they belong to Class III or IV. Restrictive factors varied across seasons: nitrate–nitrogen and BOD_5_ jointly affected water quality in winter, whereas TP predominantly constrained water quality in summer. These results could provide a reference for water quality monitoring and management strategies of constructed wetlands in different seasons in Jiangsu Province.

## 1. Introduction

Urban lakes serve as crucial providers of various ecosystem services and are tightly connected with human well-being. These services encompass vital functions such as flood control, drought alleviation, climate regulation, as well as the maintenance of species populations and habitats [1,2]. The rapid pace of urbanization profoundly impacts urban lakes, ushering in numerous ecological and environmental challenges that diminish their ecological service value [3]. Specifically, lake eutrophication and cyanobacterial blooms remain unabated, resulting in the degeneration of aquatic vegetation, diminishing lake purification capacities, and ultimately reducing lake biodiversity [4].

Nitrogen (N) and phosphorus (P) serve as indispensable elements in photosynthesis and plant metabolism, assuming pivotal roles in the nutrient cycling of lake ecosystems and exerting significant influence on both water quality and ecosystem dynamics [5,6]. Among them, N, serving as a key factor in the synthesis of proteins, nucleic acids, and other nitrogen-containing biomolecules, also acts as a main limiting factor for the primary productivity of lakes [7,8]. The nitrogen compounds present in water in the form of dissolved inorganic nitrogen primarily include ammonia–nitrogen, nitrate–nitrogen, and nitrite–nitrogen [9]. P is one of the major drivers of eutrophication in aquatic systems, and P exists in the form of phosphate, polyphosphate, and organic phosphorus [10]. Currently, anthropogenic disturbances that cause imbalances in N, P, and other nutrient elements can directly lead to anomalous surges in the primary productivity of lake ecosystems, thereby triggering eutrophication [11]. Consequently, the effective management of N and P inputs is widely regarded as the most efficacious strategy for controlling eutrophication in aquatic ecosystems [12].

Lake nutrient mitigation strategies encompass such approaches, which include pollution load reduction via source control, stormwater filtration, constructed wetland interception, the dredging of nutrient-rich sediments, aeration and oxidation to suppress sediment nutrient release, and the addition of chemicals for P removal [13]. Notably, methods such as chemical addition and sediment dredging prove to be time-sensitive, whereas constructed wetlands have emerged as a crucial approach for the maintenance of water quality and optimization of water bodies, attributable to their ecological friendliness, lasting efficiency, and additional benefits [14]. The constructed wetland primarily eliminates N through plant absorption, substrate filtration and adsorption, ammonia volatilization, microbial ammonification, nitrification, denitrification, and anaerobic ammonium oxidation [15]. Regarding P, the reclaimed plant matter, substrate, and sediment serve as the primary P sink [16,17].

However, constructed wetlands have many limitations due to physiological characteristics of plants, and their growth conditions affect the water quality treatment process [18]. In addition, the basic water conditions, including temperature, influent flow, hydraulic load rate, nutrient load, and hydraulic residence time are also key factors affecting the overall control and nutrient removal performance of constructed wetlands [19,20]. Apart from the influencing factors susceptible to human control or interference, all other variables are seasonally dependent. Indeed, numerous studies have demonstrated that the changes in seasons markedly impact the rates of nitrification, denitrification, and plant nutrient absorption and utilization, resulting in notable fluctuations in the water purification efficiency of constructed wetlands during different seasons [20,21].

Although many studies have investigated the seasonal variations in the N and P removal efficiency of constructed wetlands and the impacts of diverse parameters, the predominant research emphasis lies in pollutant retention and adsorption within flowing water bodies, with limited exploration into the underlying mechanisms driving seasonal effects on distinct water layer segments [22,23]. Building upon prior research, this study will examine and assess water quality indicators at various depths within distinct parts of Jinshan Lake. The objectives of this study are as follows: (1) to elucidate the seasonal impacts of constructed wetlands on the diverse nutrient forms at different water depths and the underlying mechanisms; (2) to provide guidance for the management of constructed wetlands in the future, to aid decision-makers in sustainably managing lake resources.

## 2. Materials and Methods

### 2.1. Site Description

**Figure 1:**
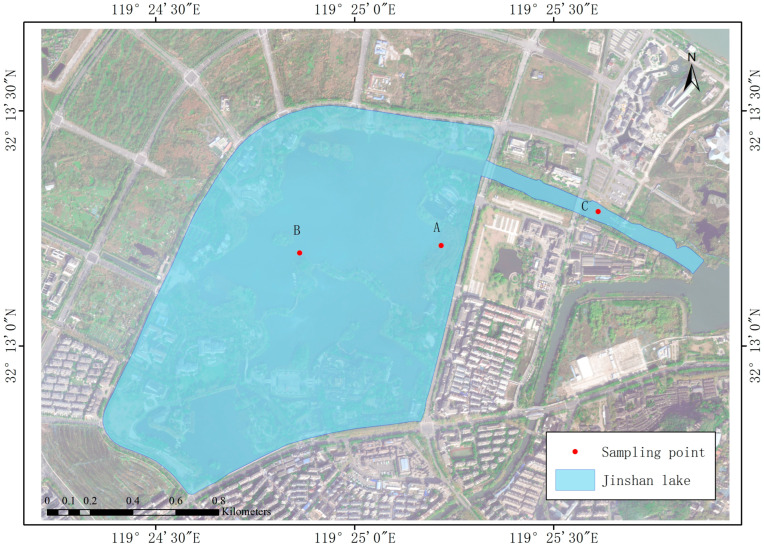
Sampling site layout in Jinshan Lake.

The study area was located in the center of Jinshan Lake, an urban reservoir of Zhenjiang City, Jiangsu Province, China (32°12′40″–32°13′28″ N, 119°24′23″–119°25′52″ E). It is a typical gate- and dam-type water body at the core of future urban development. The region features a subtropical monsoon climate, characterized by an average annual rainfall of approximately 1088.2 mm. The lake, located at 4 m above sea level, has a total area of approximately 1.08 km^2^. The water surface covers about 0.68 km^2^, which constitutes approximately 63% of the total area. Additionally, the constructed wetland spans about 20% of the total area. Taking into account the plant growth zones and the degree of human impact, field sampling was carried out in December 2022 (in winter) and June 2023 (in summer) at three specific locations—at the entrance gate C, at the constructed wetland A, and at the center of lake B—as shown in Figure 1.

Vertical water samplers were employed to collect water samples from the surface, middle, and deep layers at each of the three sites in summer and winter, respectively. The water depth varied at the different sites: the depth of water at A, B, and C was about 0.3 m, 1.75 m, and 2 m, respectively. S, M, and D represent the surface (2 cm), middle (depth/2), and deep (depth/1) water samples of each site, respectively. Samples were collected simultaneously at each sampling site three consecutive times and promptly transported to the laboratory for subsequent water sample index determination.

### 2.2. Sample Analysis and Data Processing

The measurement indexes of water in this study include chlorophyll-a (Chl-a), BOD_5_, total phosphorus (TP), total nitrogen (TN), nitrate–nitrogen, nitrite–nitrogen, ammonia–nitrogen.

The Chl-a was determined according to the “Water quality—Determination of chlorophyll a—Spectrophotometric method” [24]. BOD_5_ was determined by the “Water quality—Determination of biochemical oxygen demand after 5 days (BOD_5_) for dilution and seeding method” [25]. The TN and TP were measured after oxidation by alkaline potassium persulfate and potassium persulfate, respectively [12]. Nitrate–nitrogen was assessed through the “Water Quality—Determination of Inorganic Anions (F^−^, Cl^−^, NO_2_^−^, Br^−^, NO_3_^−^, PO_4_^3−^, SO_3_^2−^, SO_4_^2−^)-Ion Chromatography” [26]. Nitrite–nitrogen was determined according to the “Water quality—Determination of nitrogen (nitrite)-Spectrophotometric method” [27]. Ammonia–nitrogen was determined by “Water quality―Determination of ammonia nitrogen―Nessler’s reagent spectrophotometry” [25].

LSD tests and Welch’s analysis of variance were performed to identify the data groups that characterize differences. The correlation between two variables was measured by performing Pearson’s correlation analysis. All data were processed using SPSS 25.0. Plotted images were generated using Origin 2018. In addition, the water quality indicators were assessed in accordance with the “Environmental Quality Standards for Surface Water” [28].

### 2.3. Water Quality Evaluation Methods

In order to strictly and fully reflect the water quality and promptly determine its category and any deviations from standards, this paper employs the single-factor evaluation method for evaluating water environment quality. The single-factor evaluation method compares all evaluated indicators with the limit values specified in the “Surface Water Environmental Quality Standards”, selecting the class corresponding to the poorest indicator as the comprehensive water quality category of the water [28].

The calculation method for the single-factor index of water quality can be expressed as follows:

$$\;G={\left(G_{i}\right)}_{MAX}$$

where *G* is the comprehensive water quality classification; *G_i_* is the classification of the parameter *i*; (*G_i_*)*_MAX_* is the maximum classification for all of the parameters.

According to the “Environmental Quality Standards for Surface Water”, the basic project standard limits for surface water environmental quality are outlined in Table 1. In the standard, Class I is mainly for the sources of water and national nature protection areas; Class II is mainly for Class I protection areas for centralized potable water sources, protection areas for rare fishes, spawning grounds for fishes and shrimps, etc.; Class III is mainly for Class II protection areas for centralized potable water sources, protection areas for general fishes and swimming areas; Class IV is mainly for general industrial water areas and entertainment water areas; Class V is mainly for farmland water areas and water areas for general landscape requirement. Water quality below Class III is no longer suitable for drinking and water quality below Class V does not support aquatic ecosystem health [29].

## 3. Results

### 3.1. Chlorophyll a

**Figure 2:**
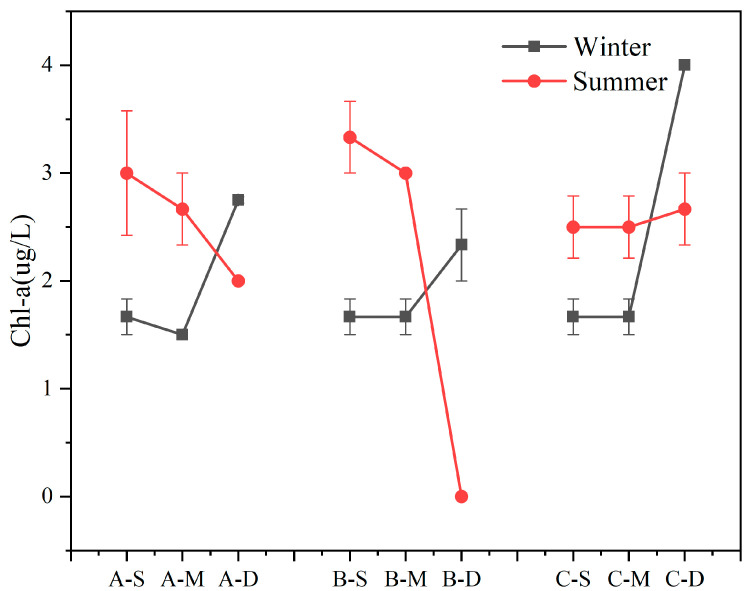
Content of Chl-a in summer and winter (values are means, n = 3; error bars represent standard error).

The seasonal variations and vertical distributions of Chl-a concentration in various regions are presented in Figure 2. Overall, Chl-a concentration in the study area exhibited significant depth-dependent variations, a decrease with water depth in summer and an overall increase in winter were observed. During winter, Chl-a concentration in the bottom layer surpassed that of the surface- and middle-layer water samples (n = 27, *p* < 0.01). During summer, the concentration of Chl-a in the bottom layer was lower than that in the surface and middle layer, except at site C (n = 18, *p* < 0.01). Furthermore, compared to the average Chl-a concentration of the three samples, site B exhibited the highest value in summer and the lowest value in winter, resulting in the largest disparity between the average summer and winter concentrations among the three samples, reaching 1.0 μg L^−1^. During winter, the highest Chl-a concentration was detected in the bottom water body at C, reaching 4.0 μg L^−1^.

### 3.2. Biochemical Oxygen Demand after 5 Days

**Figure 3:**
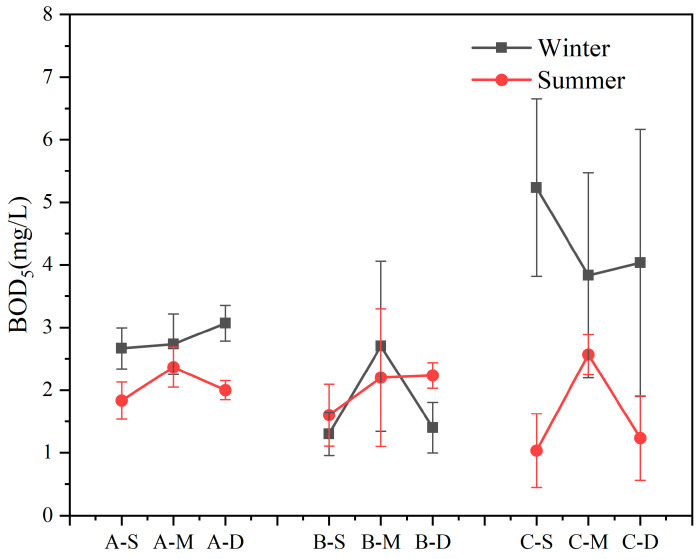
Content of biochemical oxygen demand after 5 days (BOD_5_) in summer and winter (values are means, n = 3; error bars represent standard error).

Within the study area, BOD_5_ exhibited erratic fluctuations with variations in water sample depth in both seasons (Figure 3). Apart from site B, BOD_5_ content was higher in winter compared to summer (n = 18, *p* < 0.05). Notably, at site C, BOD_5_ content in the winter surface layer exceeded that in summer by 5.08 times, with a recorded maximum concentration of 5.23 mg L^−1^. Overall, except at site C in winter, there were no significant differences in BOD_5_ concentrations at other sites.

### 3.3. Nitrogen

**Figure 4:**
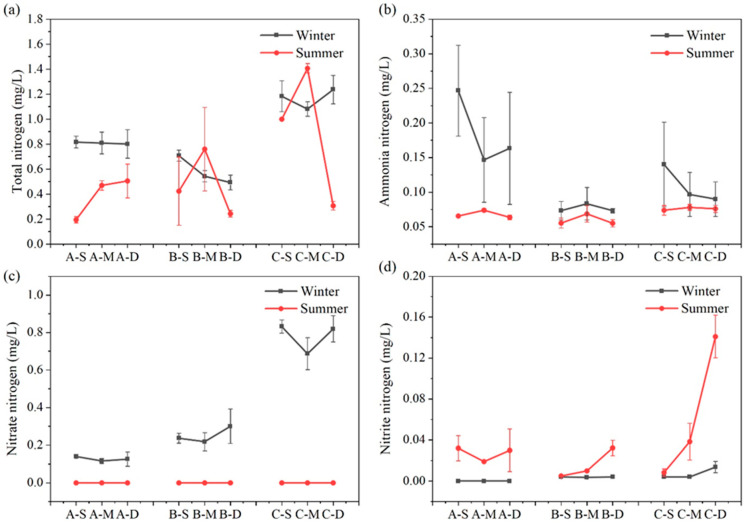
Content of TN (**a**), ammonium–nitrogen (**b**), nitrate–nitrogen (**c**), and nitrite–nitrogen (**d**) at different depths in summer and winter at three sites (values are means, n = 3; error bars represent standard error).

The seasonal variation and vertical distribution of different N forms in each region are depicted in Figure 4. Overall, TN content in each water layer of the constructed wetland remained relatively stable across seasons. Concerning various nitrogen forms, concentrations of ammonia–nitrogen, nitrate–nitrogen, and nitrite–nitrogen exhibit pronounced seasonal variations. During summer, the concentrations of ammonia–nitrogen and nitrate–nitrogen in the surface, middle, and bottom water samples were consistently lower compared to winter (n = 27, *p* < 0.01); conversely, concentrations of nitrite–nitrogen are significantly higher during summer than winter (n = 27, *p* < 0.01).

The concentration of TN exhibits no discernible trend with water depth. During winter, the concentration of TN at the three sampling sites follows the sequence C > A > B (n = 3, *p* < 0.05), whereas in summer, it is C > B > A (n = 3, *p* < 0.05). In summer, the middle water sample at C has the highest TN concentration, reaching 1.41 mg L^−1^, while the surface water sample at A has the lowest TN concentration, only 0.20 mg L^−1^. During summer, the ammonia–nitrogen concentration of the three sites increased first and then decreased with the water depth. In winter, the ammonia–nitrogen concentration trend at site B mirrored that of summer, whereas at sites A and C, it decreased initially before increasing. The highest ammonia–nitrogen concentration, reaching up to 0.25 mg L^−1^, was observed in the surface water of constructed wetland A during winter, significantly surpassing that of other water samples. Nitrate–nitrogen content in summer fell below the detection limit, while during winter, all sites exhibited a trend of initially decreasing and then increasing with water depth. In winter, compared with the average nitrate–nitrogen concentration in the three sites, C > B > A, the average nitrate–nitrogen concentration at C was 6.09 times that at A. During summer, the nitrite–nitrogen concentration at site A initially decreased before increasing, while at sites B and C, it increased with water depth. In addition, the nitrite–nitrogen concentration of bottom water at site C in summer reached the maximum, up to 0.14 mg L^−1^.

### 3.4. Distribution of N Content and Proportion of Each Form

**Figure 5:**
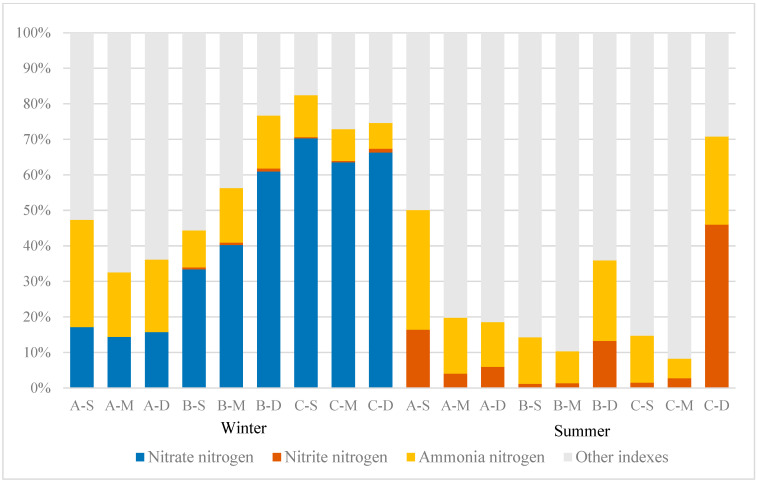
Proportions of different nitrogen forms.

The distribution of N content and proportions in various forms in the study area can reflect the relative contributions of different nitrogen components to TN, as illustrated in Figure 5. In this study, the proportion of nitrate–nitrogen and nitrite–nitrogen in TN exhibits a pronounced seasonal variation. Throughout winter, nitrate–nitrogen accounts for a significant proportion. At sites A, B, and C, the proportions of nitrate–nitrogen range from 14.40% to 17.14%, 33.4% to 60.96%, and 63.52% to 70.25%, respectively. Ammonia–nitrogen accounts for proportions ranging from 18.13% to 30.20%, 10.36% to 15.36%, and 7.28% to 11.83% at these locations, respectively. Nitrite–nitrogen accounts for a minimal proportion, never exceeding 2% of the TN. Conversely, during summer, the proportion of nitrate–nitrogen plummeted to nearly zero, while the proportion of nitrite–nitrogen increased significantly. Specifically, the proportions of nitrite–nitrogen in areas A, B, and C ranged from 4.04% to 16.41%, 1.18% to 13.29%, and 1.49% to 45.98%, respectively.

The main depths at which nitrite–nitrogen predominantly exists vary among the three sampling sites. At site A, nitrite–nitrogen predominantly accumulated near the water surface, constituting 62.18% of the total site concentration, whereas at sites B and C, it primarily concentrated near the bottom, comprising 84.18% and 91.61%, respectively. Regarding ammonia–nitrogen, the proportions at sites A, B, and C ranged from 12.61% to 33.68%, 9.04% to 22.60%, and 5.55% to 24.78%, respectively. It is evident that the proportion of ammonia–nitrogen in constructed wetland A was notably higher than that at sites B and C during both summer and winter. During summer, nitrogen primarily existed in other forms, such as organic nitrogen and free nitrogen.

### 3.5. Phosphorus

**Figure 6:**
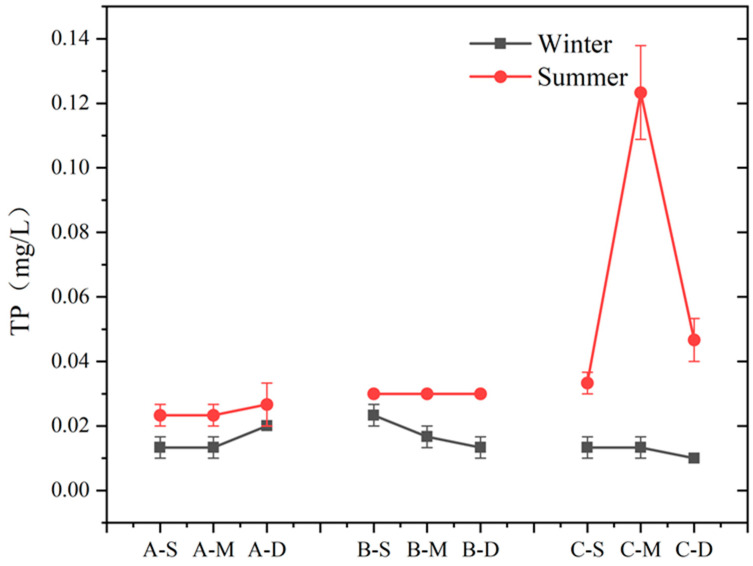
Content of TP at different depths in summer and winter at three sites (values are means, n = 3; error bars represent standard error).

The seasonal variation and vertical distribution of TP in each region are depicted in Figure 6. During summer, TP concentration at sites A and B exhibited minimal variation with water depth, whereas at site C, it initially increased and then decreased with depth. In winter, there was no significant vertical difference in TP concentration among the three sites. In this study area, TP concentrations differed significantly between summer and winter, with higher concentrations observed during summer compared to winter (n = 9, *p* > 0.05). Notably, at the middle layer of site C, TP concentration in summer was ten times higher than in winter, reaching 0.10 mg L^−1^. During winter, the difference in TP content was less pronounced.

### 3.6. Correlation Analysis

**Figure 7:**
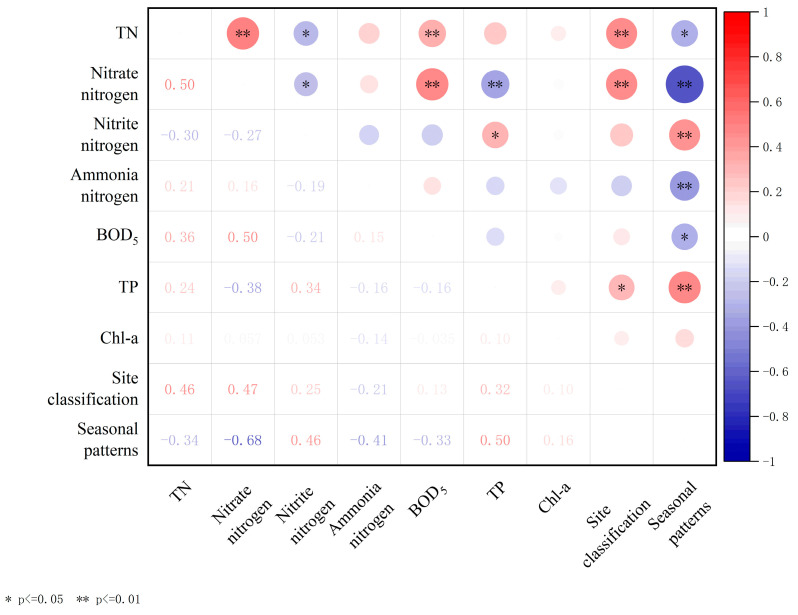
Heatmap of Pearson’s correlation matrix for total nitrogen (TN), total phosphorus (TP), and other indexes in water of Jinshan Lake.

This study conducts correlation analysis on various variables, including TN, nitrate–nitrogen, nitrite–nitrogen, ammonia–nitrogen, BOD_5_, TP, and Chl-a, and displays a heatmap of Pearson’s correlation matrix (Figure 7). The analysis results show that BOD_5_ exhibited significant positive correlations with TN and nitrate–nitrogen contents (r = 0.36, *p* < 0.01; 0.50, *p* < 0.01). In addition, there was a noteworthy positive correlation between the nitrate–nitrogen content and TN content (r = 0.50, *p* < 0.01). Additionally, examination of Figure 3 and Figure 4 reveals a similarity in the trends of variation among these three potential pollutants in aquatic environments, implying a robust correlation in their influencing factors and transformations. Seasonal patterns exhibit noteworthy positive correlations with nitrite–nitrogen and TP (r = 0.46, *p* < 0.01; r = 0.50, *p* < 0.01), alongside significant negative correlations with nitrate–nitrogen and ammonia–nitrogen (r = −0.68, *p* < 0.01; r = −0.41, *p* < 0.01). Site classification exhibits a significant positive correlation with TN and nitrate–nitrogen (r = 0.46, *p* < 0.01; r = 0.47, *p* < 0.01).

### 3.7. Water Quality

Recognizing that the single-factor assessment method not only directly delineates the water quality classification but also reflects the standards, this study employs said method to assess Jinshan Lake’s water quality in accordance with the “Environmental Quality Standards for Surface Water” [28]. The monitored water quality parameters encompass TN, TP, ammonia–nitrogen, and BOD_5_. The evaluation grades for each parameter and the assessment outcomes for each sampling site are delineated in Table 2.

As indicated in Table 2, the three sampling sites experienced varying degrees of pollution during both seasons, with monitoring results indicating the water quality are Classes III and IV. TN and TP, key nutrients contributing to lake eutrophication, exhibit seasonal variations in surpassing standard levels, with TN primarily exceeding standards in winter and TP mainly surpassing standards in summer. During winter, B demonstrates relatively good water quality, whereas during summer, A exhibits comparatively better conditions, meeting Class II water quality standards in the surface and middle layers, despite elevated TP levels in its bottom layer. Conversely, water quality at C is consistently rated Class IV throughout both seasons, with BOD_5_ exceeding standards in winter. Overall, BOD_5_ levels show a seasonal pattern of lower levels in summer and higher levels in winter. Vertically, apart from notable disparities in water quality at A in summer, minor variations are observed at other sites in both winter and summer.

## 4. Discussion

### 4.1. Seasonal Variations in the Main Pollutants of Water (N and P) and the Function of Constructed Wetlands

The concentrations of N and P serve as crucial indicators of water eutrophication [30]. Generally, constructed wetlands effectively intercept, transform, and remove nutrients like N and P from water, particularly during summer [31]. Nevertheless, the results indicate that N and P concentrations in Jinshan Lake water remain elevated (Table 2). During summer, TN levels are elevated at site C, particularly in the middle water layer (Figure 4a). Farmland runoff upstream of site C may serve as the primary pollutant source. Following the passage through site A, there was a notable 56.7% reduction in the mean TN value, indicative of a substantial alteration in TN concentration. This observation suggests that the vegetation within the constructed wetland effectively facilitated the absorption of N from the water. Nonetheless, during winter, the efficiency of nutrient removal by A diminishes due to decreased microbial activity and plant biomass at lower temperatures, resulting in notably lower TN removal compared to summer. The removal efficiency of TN in constructed wetlands is subject to seasonal variations [23]. Nitrogen is present in diverse forms within water, encompassing nitrate–nitrogen, nitrite–nitrogen, ammonia–nitrogen, and organic nitrogen [32]. Previous studies have shown that regardless of the season or water layer depth, there are different ways and pathways to affect each form of N [33,34]. The distribution of N content and its proportions in Jinshan Lake (Figure 5) also shows seasonal variations in nitrate, nitrite, and ammonia–nitrogen.

Ammonia–nitrogen, being the most reduced form of N in water, demands minimal energy during transport and assimilation, making it the favored N source for most plankton with high bioavailability [35,36]. Furthermore, previous research indicates that aside from being utilized by phytoplankton, the process of ammonia–nitrogen absorption in water involves its assimilation and absorption by heterotrophic bacteria as well as nitrification by nitrifying bacteria [37]. It is speculated that the plankton biomass of surface water and the microbial biomass of the water–sediment interface are higher in summer, which leads to the phenomenon that the ammonia–nitrogen content of surface and bottom water samples in summer is lower in the study area [38,39]. Obviously, the seasonal impact on ammonia–nitrogen is notable. A significant negative correlation exists between ammonia–nitrogen content and seasons in Jinshan Lake (*p* < 0.01). The ammonia–nitrogen content is higher in winter than in summer at the three sites, mirroring findings by Zhang regarding low summer concentrations due to rapid ammonia–nitrogen cycling [39]. Consequently, stricter control of ammonia–nitrogen in winter is warranted during water quality management.

Nitrate–nitrogen presents in lake water and holds significant importance as a predominant N form therein. Denitrification is the main removal mechanism, markedly influenced by seasonal variations [40,41,42]. In the study area, nitrate–nitrogen content accounted for 70.25% of TN in winter, exhibiting considerable variation among sampling sites A, B, and C, whereas it approached zero during summer. This suggests pronounced seasonal fluctuations and spatial heterogeneity in the nitrate–nitrogen content across all water samples. During winter, when the water flows from C to A, the average concentration of nitrate–nitrogen in the water decreases by 83.6%. It can be seen that the constructed wetland has a certain effect on the removal of nitrate–nitrogen from the water in winter, and this conclusion is reflected in various constructed wetlands [43,44,45]. In addition, the correlation between site classification and individual water quality indexes in this study (Figure 7) can also indicate the impact of constructed wetlands on TN and nitrate–nitrogen removal from water. The results indicate a significant correlation (*p* < 0.01) between the presence of constructed wetlands and the content of TN, particularly with N present in the form of nitrate–nitrogen. It can be seen that the removal efficiency of constructed wetlands on TN primarily manifests through its influence on nitrate–nitrogen. Besides external inputs, a portion of the nitrate–nitrogen in water originates from the nitrification of sediment organic nitrogen [46,47]. Nitrate–nitrogen content in the study area initially decreased and then increased with water depth at the three sites. The elevated concentration observed at the bottom of the water corroborates the sediment’s contribution to the aquatic environment. Therefore, to reduce the input of sediments, it is imperative to rigorously control nitrate–nitrogen content at the water inlet during winter while minimizing disturbance to the aquatic sediment.

Nitrite–nitrogen serves as an intermediary product within the N cycle’s oxidation–reduction pathways. Aquatic environments can yield nitrite–nitrogen under both aerobic and anaerobic conditions: aerobic conditions facilitate the conversion of ammonia to nitrite–nitrogen, which subsequently oxidizes to nitrate–nitrogen, whereas anaerobic conditions prompt denitrification of nitrate–nitrogen into nitrite–nitrogen, ultimately resulting in N_2_ [48]. Therefore, the accumulation of nitrite–nitrogen in water bodies hinges on the equilibrium between formation and conversion rates during nitrification and denitrification processes. In this study, nitrite–nitrogen content is notably low during winter but significantly escalates during summer, underscoring a marked seasonal fluctuation in its accumulation. Furthermore, additional correlation analysis (Figure 7) reveals a significant positive correlation between the nitrite–nitrogen content and seasons (*p* < 0.01). Previous studies have revealed that low temperatures weaken nitrate–nitrogen accumulation, which is very limited below 20 °C, until above 20 °C, when the concentration of nitrate–nitrogen accumulation increases significantly with increasing temperature [49,50]. Consequently, the effect of season on nitrite–nitrogen accumulation is modulated through water temperature, with nitrite–nitrogen accumulation increasing in summer due to higher water temperatures and the opposite in winter. Previous studies have shown a strong linear relationship between light and ammonia oxidation inhibition. It is highly probable that light causes damage to key ammonia oxidases and reduces nitrite nitrogen levels. The light intensity at the bottom of the water in the study area was weak and nitrite nitrogen was affected to a lesser extent [51]. Therefore, vertically, nitrite–nitrogen tends to accumulate more in the bottom layer of water, especially at B and C in summer.

Phosphorus removal pathways in water primarily involve plant absorption, microbial activity, and matrix adsorption [52]. Previous research has demonstrated that the presence of plants affects P concentration, and wetland plants can reduce P from the surrounding water and matrix, consequently mitigating P levels in lake water [53]. In addition to plant absorption, the removal of P from water also depends on the adsorption of the matrix. Other studies have shown that the matrix absorption is dominant in normal pores and P concentration in lake water [54]. In this study, the concentrations of P at sites A and B did not differ by the presence of plants (Figure 6), affirming the limited contribution of plants to P removal in water. Superficially, plants in constructed wetlands exert minimal influence on water surface P; however, TP content in the deep water of constructed wetlands surpassed that of other layers. Considering prior research, it can be inferred that plants primarily uptake P from sediments or substrates via roots, with their capacity for P absorption in water being limited [55,56]. Alternatively, during winter, decaying large vegetation can act as a P source within the lake, releasing a substantial amount of P into the water. This is evidenced by the elevated P concentration in the bottom layer of constructed wetlands during winter [54]. Thus, comprehensive harvesting prior to winter can significantly affect the maintenance of P levels during the season. Overall, there is a marked disparity in TP concentrations between summer and winter. Furthermore, additional correlation analysis (Figure 7) reveals a notable positive correlation between TP content and seasons (*p* < 0.01). Other research indicates that the P removal efficiency in constructed wetlands remains consistent across various seasons [57]. The generally elevated TP content at site C during summer compared to winter implies that the disparity between seasons in this study stems mainly from excessive summer inputs. Overall, this phenomenon is partly attributed to external pollution inputs or severe violations of inlet water quality standards, suggesting that human activities mainly influence seasonal TP differences in Jinshan Lake.

### 4.2. Analysis of Other Water Environment Quality Characteristic Indexes and Their Internal Relations

Chl-a, as the primary photosynthetic pigment in plants, serves as a crucial indicator for assessing algal biomass, primary productivity, and eutrophication levels [58]. Algae typically experience rapid growth under optimal nutrient, light, and temperature conditions [59,60]. The surface water of Jinshan Lake has high Chl-a concentrations due to abundant light in summer. Other research has demonstrated that Chl-a concentration is significantly influenced by seasonal variations, primarily due to the regulation of phytoplankton photosynthesis and respiratory metabolism rates by water temperature, subsequently impacting Chl-a concentration. Consequently, under typical conditions, Chl-a concentration in water is higher during summer and lower during winter [61,62]. Nevertheless, the average Chl-a concentration in the bottom water of the three sites in the study area was higher during winter than summer, possibly because the water temperature in the bottom water of Jinshan Lake, which belongs to the subtropical monsoon climate, was higher in winter, providing favorable conditions for the growth of phytoplankton [63]. In addition, other studies have shown that the presence of certain phytoplankton communities may cause Chl-a to reach local peaks at specific depths (such as below the thermocline), so the phenomenon in Jinshan Lake may also be due to the influence of phytoplankton [64].

Biochemical oxygen demand after 5 days serves as an indicator of organic matter pollution in water. Its removal primarily occurs through microorganism adsorption and metabolism, with higher values indicating increased organic pollutants and heightened pollution severity [65]. Removal efficiency is directly linked to the abundance and activity of microorganisms. Elevated temperatures stimulate microorganism proliferation, consequently enhancing the removal efficiency of BOD_5_ [66,67]; however, the seasonal variation shows relatively lower levels in summer and higher levels in winter (Figure 3). The significant presence of organic matter at site C suggests external pollution inputs during winter.

The correlation analysis of the aforementioned water indicators reveals a significant association between BOD_5_ and TN, as well as nitrate–nitrogen content (Table 2). Furthermore, nitrate–nitrogen exhibits a significant correlation with TN content. This suggests that the impact of constructed wetlands on N dynamics within lakes may primarily occur through nitrate–nitrogen content modulation.

### 4.3. Limitations and Future Development

Although the construction and maintenance of the constructed wetlands is a large investment, it can bring potential return on investment by reducing water treatment costs, improving water quality, enhancing landscape value, and upgrading ecological services. An effective management model is pivotal to the long-term water purification efficiency of constructed wetlands. Through this study, it was found that controlling the input of nitrate–nitrogen in winter and strengthening the control of TP in summer would bring long-term economic, social, and environmental benefits to the constructed wetland.

In this study, field sampling was adopted to study the seasonal spatial distribution of N and P in the lake. There was a lack of conditions for plant species and microbial numbers, which should be included for consideration in future studies. In addition, the constructed wetland at Jinshan Lake presents an example of typical water in Jiangsu Province. The conclusions may be limited, and more typical lakes and samples need to be studied in the future to determine similar mechanisms in more detail.

## 5. Conclusions

This study takes Jinshan Lake as an example to investigate the seasonal dynamics of water quality characteristics and examines the impact of constructed wetlands on distinct water layers. The main conclusions are as follows:

(1) Constructed wetlands significantly enhance TN removal during summer but only have a certain effect on nitrate–nitrogen removal in winter. However, they play a minor role in TP removal and even become a potential P source for lakes in winter due to the decomposition of wetland plant residues into sediments, threatening water quality safety;
(2) The seasonal variations in lake water environmental indicators are readily apparent. During winter, N, predominantly as nitrate–nitrogen, interacts with BOD_5_, emerging as the primary factor collectively constraining lake water quality. During summer, TP assumes the primary role as the constraining factor of water quality. As an intermediate product in the N cycle’s redox pathway, the accumulation of nitrite–nitrogen correlates with water temperature across varying seasons. Nitrite–nitrogen accumulation will rise with increasing water temperatures in summer, whereas the converse holds true in winter;
(3) Chl-a concentrations are higher under conditions of sufficient light and suitable temperature. Concurrently, the biomass present in the surface water layer and microbial biomass at the water–sediment interface exert substantial influence on ammonia–nitrogen removal efficiency via assimilation and nitrification within constructed wetlands. Moreover, surface water illumination damages ammonia oxidase, reducing the content of nitrite–nitrogen.

## Figures and Tables

**Table 1:** Values of surface water quality standard concentrations in China (mg/L).

Items		Class I	Class II	Class III	Class IV	Class V
BOD_5_ (mg/L)	≤	3	3	4	6	10
NH_3_-N (mg/L)	≤	0.15	0.5	1.0	1.5	2.0
TP (mg/L)	≤	0.02	0.1	0.2	0.3	0.4
TN (mg/L)	≤	0.2	0.5	1.0	1.5	2.0

Note: data from the Ministry of Ecology and Environment of the People’s Republic of China.

**Table 2:** Evaluation results of each index and results of the single-factor evaluation method.

Season		BOD_5_	NH_3_-N	TP	TN	Water Quality Classification	Excessive Factor	Grade
Winter	A-S	I	II	I	III	III	TN	III
	A-M	I	I	I	III	III	TN	
	A-D	III	II	II	III	III	TN	
	B-S	I	I	II	III	III	TN	III
	B-M	I	I	II	III	III	TN	
	B-D	I	I	II	II	II	——	
	C-S	IV	I	I	IV	IV	TN, BOD_5_	IV
	C-M	III	I	I	IV	IV	TN	
	C-D	IV	I	I	IV	IV	TN, BOD_5_	
Summer	A-S	I	I	II	I	II	——	IV
	A-M	I	I	II	II	II	——	
	A-D	I	I	III	III	IV	TP	
	B-S	I	I	III	II	IV	TP	IV
	B-M	I	I	III	III	IV	TP	
	B-D	I	I	III	II	IV	TP	
	C-S	I	I	III	III	IV	TP	IV
	C-M	I	I	IV	IV	IV	TP, TN	
	C-D	I	I	III	II	IV	TP	

## Data Availability

All data from the study are included in the article.

## Abbreviations

TN: total nitrogen; TP: total phosphorus; Chl-a: chlorophyll-a.
